# Articulation constrained learning with application to speech emotion recognition

**DOI:** 10.1186/s13636-019-0157-9

**Published:** 2019-08-20

**Authors:** Mohit Shah, Ming Tu, Visar Berisha, Chaitali Chakrabarti, Andreas Spanias

**Affiliations:** 1 School of Electrical, Computer, and Energy Engineering, Arizona State University, Tempe, AZ USA; 2 Speech and Hearing Science Department, Arizona State University, Tempe, AZ USA

**Keywords:** Emotion recognition, Articulation, Constrained optimization, Cross-corpus

## Abstract

Speech emotion recognition methods combining articulatory information with acoustic features have been previously shown to improve recognition performance. Collection of articulatory data on a large scale may not be feasible in many scenarios, thus restricting the scope and applicability of such methods. In this paper, a discriminative learning method for emotion recognition using both articulatory and acoustic information is proposed. A traditional *ℓ*_1_-regularized logistic regression cost function is extended to include additional constraints that enforce the model to reconstruct articulatory data. This leads to sparse and interpretable representations jointly optimized for both tasks simultaneously. Furthermore, the model only requires articulatory features during training; only speech features are required for inference on out-of-sample data. Experiments are conducted to evaluate emotion recognition performance over vowels */AA/*, */AE/*, */IY/*, */UW/* and complete utterances. Incorporating articulatory information is shown to significantly improve the performance for valence-based classification. Results obtained for within-corpus and cross-corpus categorical emotion recognition indicate that the proposed method is more effective at distinguishing happiness from other emotions.

## Introduction

The study of speech-based emotion recognition has gained practical importance with the recent advances in human-machine interaction. Emotions convey important information related to the speaker’s mood or personality originated from neural activities [1], which can be used to assist and improve the performance of automatic speech recognition or spoken dialog systems [2, 3]. This topic has further applications in automated call centers [4], child education [5], entertainment, patient care, and diagnosing post-traumatic stress disorders [6]. Emotion recognition is a challenging task since their expression and perception vary greatly across speakers, cultures, and languages. Often, information is required from multiple sources to form a reliable estimate of emotion [7–10].

**Fig. 1:**
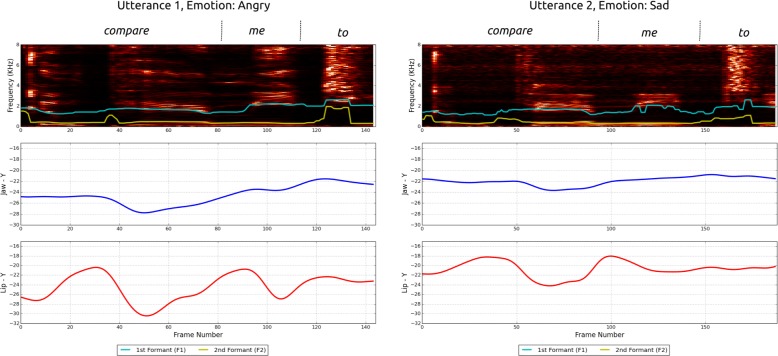
Relationship between angry (left panel) and sad (right panel) emotions, acoustic characteristics, and articulatory information for an utterance *“compare me to”* by a male speaker. (Top row) Spectrogram and formant tracks, (middle row) position of the jaw along *Y*-axis, and (bottom row) position of the lip along *Y*-axis. Note the differences in articulatory position and frequency response for vowels */AE/* and */IY/* of words *“compare”* and *“me”*, respectively. Negative axis corresponds to downward movement along the *Y*-axis

Emotional speech can be characterized by various descriptors—acoustic properties, spoken content, and articulatory position/movement. The majority of research is focused on using the acoustic properties of speech, owing to their strong correlations with emotion and simple recording procedures. However, it is commonly understood that articulator motion also exhibits a strong correlation with emotion. One such example highlighting this relationship between emotions, articulatory movement, and acoustic characteristics is depicted in Fig. 1. Here, for the vowel */AE/* in the word *compare*, anger forces a larger opening of the jaw as opposed to sadness. Similarly, lips are more protruded towards the outside for the vowel */IY/* in the word *me* under anger. The differences in articulatory movement also correspond to speech spectral differences between anger and sadness [11–13]. Below, we provide an overview of the existing work on emotion recognition and related applications that make use of articulatory kinematics.

There is a vast literature devoted to studying emotion recognition using acoustic features [14–18]. Low-level descriptors (LLDs) of the spectral or prosodic type, such as Mel filter banks (MFBs), pitch, or intensity, have been used in [17, 19–22]. Supra-segmental representations derived over linguistic units such as phonemes, words, or sentences have proven to be more successful than frame-based or instantaneous segmental approaches [14, 23, 24]. Supra-segmental features are typically obtained by calculating various statistics from the LLDs over the defined linguistic unit. The size of the feature set often ranges from as few as 100 to as high as 5000, depending on the number of statistics extracted [16, 23, 25]. Discriminative classifiers are then trained over such high-level features for a multitude of tasks such as binary (arousal, valence) or categorical (happy, sad, angry, etc.) classification as well as regression over continuous emotional attributes [26–31]. Recently, deep neural network (DNN)-based models trained on simple frame-level speech features have achieved better performance than models trained on human-engineered utterance-level features [32–35]; however these models typically require training on large, labeled data sets.

On the other hand, there are relatively few studies that attempt to characterize emotion using articulatory information. In [11], it was shown that the degree of jaw opening increased significantly as subjects became annoyed, while in [12], the lateral lip distance between the corners of the mouth was shown to be strongly influenced by the emotional state. In [13], the authors showed that articulation-based features achieved a much better classification rate compared to acoustic features for a single male subject. Articulatory data in each of the above works was collected using a multi-channel electromagnetic articulography (EMA) system. Alternatively, articulatory data captured using facial markers have been applied in a multi-modal framework [7, 36]. In [36], the authors showed that the lower region of the face (chin and lips) was the best indicator of emotion. These studies are mostly limited to single subjects, or multiple speakers recorded under similar conditions. However, more importantly, these methods require articulatory data during inference on out-of-sample data in order to perform reliably. Acquisition of such data on a large scale is difficult and time-consuming due to its invasive and highly sensitive recording procedure; this limits the scope and application of these methods.

To alleviate the need for articulatory data during training, several studies propose to use pre-trained acoustic-to-articulatory inversion models to first estimate articulatory features and then to use these features in different speech classification tasks [37]. For example, the authors in [38] evaluated the utility of articulatory features *estimated* from a pre-trained acoustic-to-articulatory inversion model for emotion recognition. In [39], a similar approach is used for phonetic classification. The authors in [40] integrated the acoustic-to-articulatory mapping system within a DNN architecture and used this architecture for both senone classification and feature mapping. The study in [41] augmented input acoustic features with estimated articulatory features generated from a pre-trained acoustic-to-articulatory model. This body of work suggests that augmenting speech classification models with estimated articulatory features yields improved performance; however, these approaches rely on complex and non-linear acoustic-to-articulatory models that are not optimized in any way for the underlying task of interest.

In this paper, we propose a discriminative learning method for emotion recognition using articulatory and acoustic information. Rather than training a stand-alone acoustic-to-articulatory inversion model, the proposed articulation constrained learning (ACL) method is formulated to *jointly* minimize emotion classification error and articulatory reconstruction error using articulatory and acoustic features from the same or different databases. Compared to the previous work in [38–41], this results in a much simpler model that is specifically optimized for emotion recognition. We evaluate the model by extending the conventional logistic regression cost function to include additional constraints that enforce the model to also reconstruct articulatory data. The classifier weights are constrained to be sparse via L1 regularization, which leads to a shared and interpretable representation. We note that the method is not limited to linear models only. The articulation reconstruction criterion can be used to constrain DNN learning through regularization; however, in this paper, we focus on a linear model given the limited size of the training set and leave the DNN extension for future work.

The proposed regularizer in ACL is well suited for databases with high dimensional feature sets and limited articulatory data, as is common in emotion recognition studies. A review of 32 publicly available speech emotion databases reveals that most are comprised of a very small number of subjects with, at most, a few hundred instances per class [42]. Since our method constrains the solution space during training by requiring that the learned model focuses on acoustic features that capture articulatory kinematics, it has similarities with the literature on using transfer learning for emotion recognition [43–45]. In transfer learning, the aim is to improve performance on a target task (emotion recognition) by transferring the shared knowledge that exists in a related source task (articulator motion reconstruction). This is especially useful in applications where training data is limited but the feature dimension is large [43, 46]. In the literature, transfer learning is typically used as a means of adapting the model to new domains by including limited data from the source domain during training (e.g., new background noise conditions and new speakers) [45]. Our application of transfer learning is quite different; our aim is to use the articulator reconstruction task to improve emotion recognition models. In contrast to this previous work, the contributions of our work can be summarized as follows:

- A speaker-independent, articulation-constrained learning model is proposed, where the hypothesis space for the emotion recognition model is constrained by requiring that the model jointly predicts emotion and articulator kinematics. In this paper, the model is derived for a linear discriminative learning example; however, the method can be extended to other models, including DNNs;
- The resultant model is much simpler compared to other approaches that estimate articulatory information using complex acoustic-to-articulatory inversion models;
- In contrast to most multi-modal emotion recognition methods, articulatory data is not required during inference;
- We conduct cross-corpus studies, where the acoustic features used for emotion recognition and articulatory target reconstruction belong to different databases.

Experiments are performed using two databases, the USC EMA [13] and the USC IEMOCAP [47], consisting of acted and scripted emotional utterances. Earlier studies [13, 48] have shown that the dependence between emotions, acoustic characteristics, and articulatory kinematics is clearly manifested in the vowels. Hence, the performance is first evaluated over 4 peripheral vowels, such as */AA/*, */AE/*, */IY/*, and */UW/*. Additional experiments are conducted to evaluate the performance over complete utterances, involving all vowels and consonants. Overall, the proposed method shows significant improvements in valence classification over traditional approaches based on acoustic features only. Further experiments on categorical emotion recognition show that articulatory information helps improve the accuracy of recognizing happy emotions; this is important for applications that evaluate how emotion spreads in group settings and its impact on productivity [49]. In a mixed-corpus scenario in which the speech data comes from one corpus and the articulatory data comes from another corpus, the performance is observed to be almost similar to a within-corpus scenario, which highlights the ability of the proposed method to generalize well across databases with speech and articulatory information recorded using different techniques and under different conditions.

The remainder of this paper is organized as follows: The databases used in this work are described in Section 2. The proposed ACL method is described in Section 3. Experiments and results for within- and cross-corpus experiments are presented in Sections 4 and 5, respectively. Finally, conclusions are presented in Section 7.

## Data preparation

A brief overview of the two databases, USC EMA and USC IEMOCAP, and the respective articulatory information are described in this section.

### The USC EMA database

The USC EMA database is comprised of scripted and acted emotions by three (1 male, 2 female) speakers [13]. A set of 14 sentences, mostly neutral in emotional content, were used. Four different emotions, i.e., neutral, angry, sad and happy, were simulated by each speaker. The male speaker recorded each sentence 5 times for each emotion resulting in a total of 280 utterances. The female speakers performed the same exercise, with only 10 out of 14 sentences, resulting in 200 utterances per speaker. From a total of 680 utterances, only those utterances were chosen for which external evaluators were in consensus with regard to the perceived emotion, resulting in a set of 503 utterances.

Articulatory data was collected using an EMA system. The positions of three sensors attached to the tongue tip, the lower maxilla (for the jaw movement), and the lower lip are tracked. Each sensor trajectory (target) was recorded along the *X* (forward-backward movement) and *Y* (vertical movement) axes. The velocity and acceleration of each trajectory are also included in addition to the position. This results in a total of 18 articulatory targets, as shown in Table 1. Articulatory data is recorded at a sampling rate of 200 Hz, and speech is recorded at a sampling rate of 16 KHz.

**Table 1:** Articulatory targets for the USC EMA and USC IEMOCAP databases

Location	Attributes	Axes	Total
USC EMA			
Tongue (TNG)	Position (POS)		
Jaw (JAW)	Velocity (VEL)	(*X,Y*)	18
Lip (LIP)	Acceleration (ACC)		
USC IEMOCAP			
Chin (CHN)	Chin Width (CHW)	(*X,Y*,*Z*)	15
	Chin Position (CHP)		
Lip (LIP)	Lip Width (LPW)		
	Lip Height (LPH)		
	Lip Position (LPP)		

**Fig. 2:**
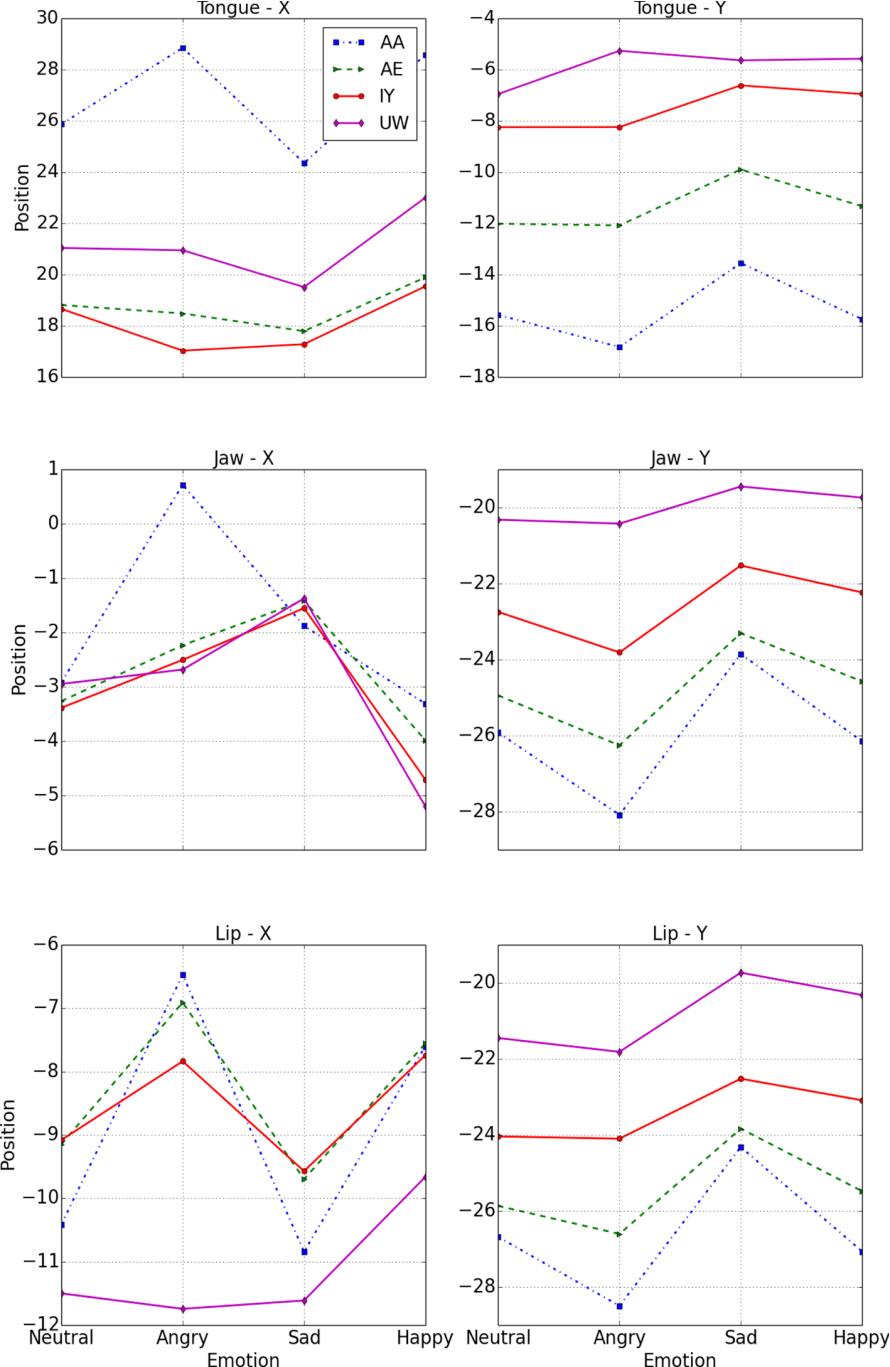
Mean position of select articulatory targets, averaged across all speakers, for different vowels and emotions in the USC EMA database. (Top row) Tongue, (middle row) jaw, and (middle row) lip. Negative axis corresponds to forward movement along *X*-axis and downward movement along *Y*-axis

Figure 2 shows the mean value of the *X,Y* coordinates for the tongue, jaw, and lip positions for different vowels and emotions. Angry utterances show the most distinct characteristics compared to other emotions. This effect is prominent especially for the vowels *AE* and *AA*. Previously, researchers studied these aspects in detail and showed that such differences are statistically significant [13]. This further highlights the dependence between emotions and articulatory kinematics.

### The USC IEMOCAP database

The USC IEMOCAP database [47] was collected by asking five pairs of male-female actors to elicit emotions either by reading from a script or via improvisation in a conversational setting. This database consists of a total of 10,039 utterances. Categorical attributes, including neutral, sad, happy, angry, frustrated, surprised, disgust, fear, and unknown, are assigned to each utterance. Only scripted utterances for which a majority consensus was reached among external evaluators are considered in this study. Only utterances labeled as neutral, sad, happy, excited, and angry are selected, while the remaining attributes are not considered as they are under-represented. Furthermore, happy and excited are treated as the same emotion and merged under one class. This results in a total of 1262 utterances distributed across ten speakers and four emotions. The procedure for data selection and labeling is similar to previous studies conducted on this database [9, 18, 24].

**Fig. 3:**
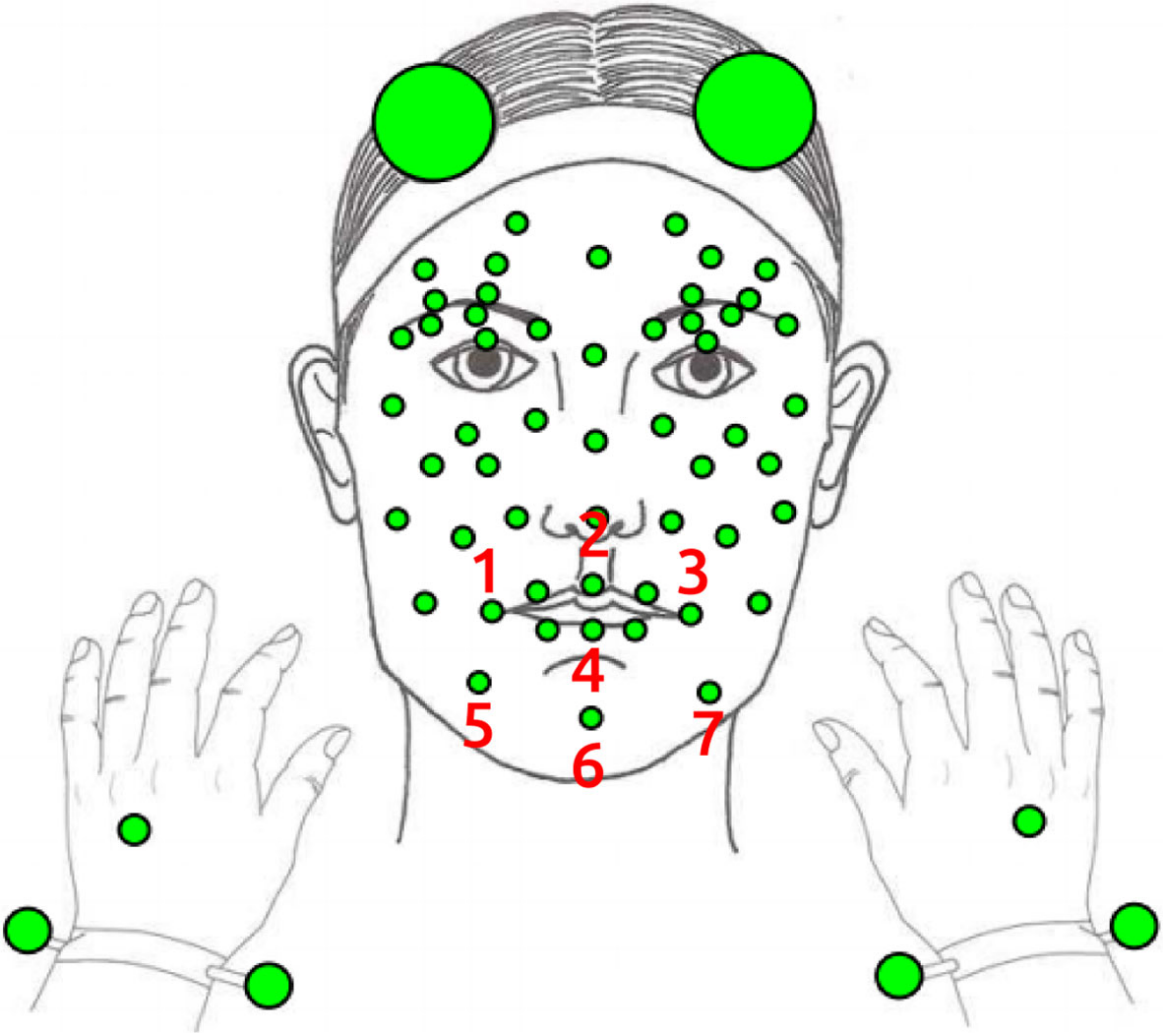
Arrangement of facial markers in the USC IEMOCAP database. The markers used in this study are numbered from 1 to 7. Image obtained from [47]

Articulatory information is available in the form of motion capture markers located at different points on a speaker’s face. An example arrangement with 53 facial markers is shown in Fig. 3. This was originally intended for studying facial expressions; hence, not all markers contain information relevant to articulation. Markers located in the chin and lip areas are considered in this study as they are expected to relate the most to articulatory movement. Specifically, the chin position (6), width of the chin (difference between 5 and 7), lower lip position (4), lip height (difference between 2 and 4), and lip width (difference between 1 and 3) are considered. Each marker is represented by its (*X,Y*,*Z*) co-ordinates, resulting in a total of 15 articulatory targets, as described in Table 1. Articulatory data is recorded at a sampling rate of 120 Hz. Speech is recorded at a sampling rate of 48 KHz and downsampled to 16 KHz prior to feature extraction.

**Fig. 4:**
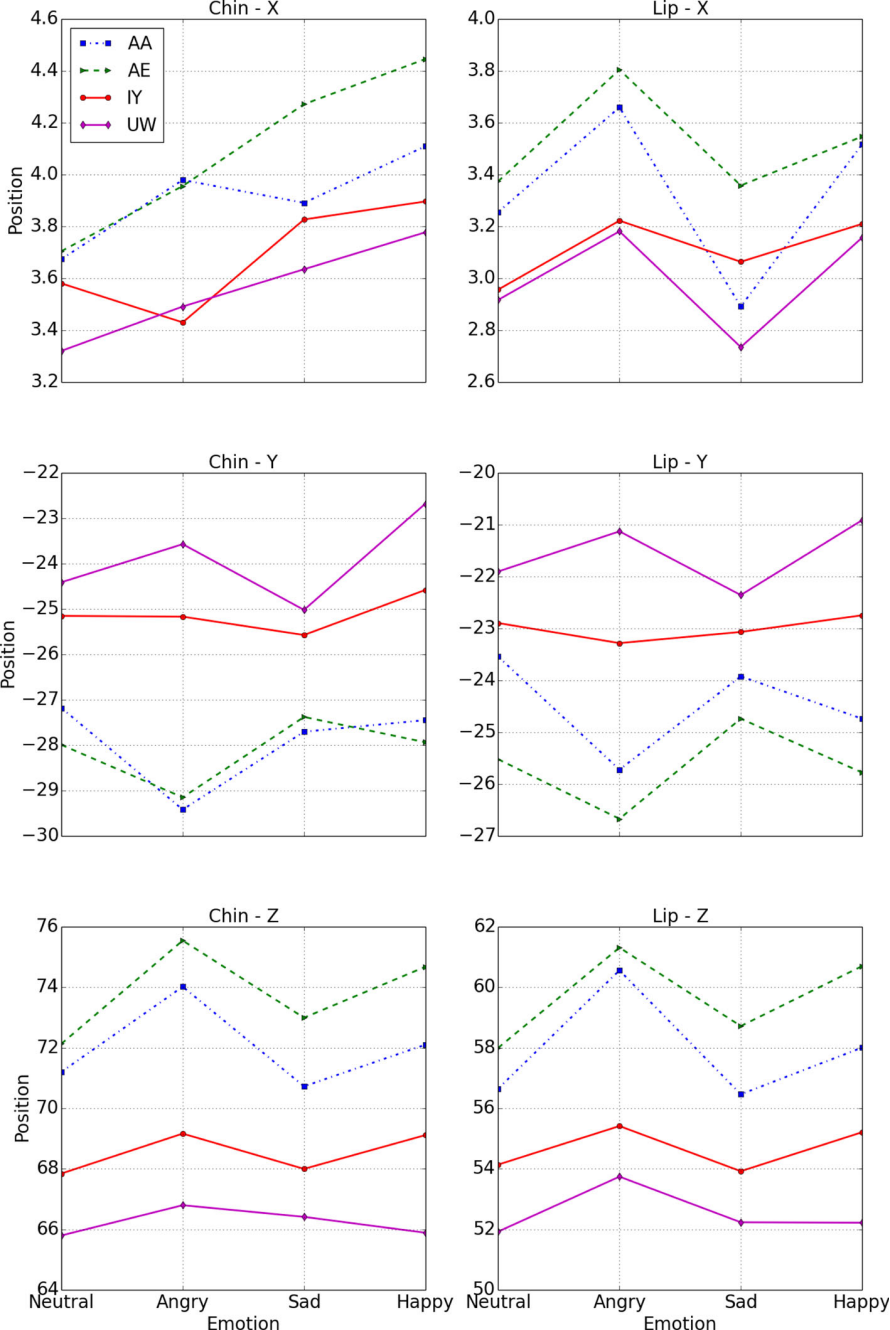
Mean position of select articulatory targets, averaged across all speakers, for different vowels and emotions in the USC IEMOCAP database. (Top row) *X*, (middle row) *Y*, and (bottom row) *Z* coordinates

Figure 4 shows the mean value of the *X,Y*,*Z* coordinates for the chin and lower lip positions, averaged across all speakers, for different vowels and emotions in the USC IEMOCAP database. There is a strong correlation between the chin and lip positions along the *Y,Z* axes. Similar to the USC EMA database, the articulatory behavior is different across emotions for the same vowel. For */UW/*, the position of the chin along the *Y*-axis for sadness is higher compared to happiness or anger. The effectiveness of these markers towards emotion recognition was studied in [36].

Comparing the two databases, USC EMA and USC IEMOCAP, a few more similarities can be observed from Figs. 2 and 4. The jaw/chin/lip position along the *Y*-axis is lower for vowels */AA/* and */AE/*, especially for anger and happiness. The behavior of the lip position along the *X*-axis is also similar across the two databases for different vowels. In comparison with the acoustic characteristics of different emotions, which vary highly across speakers and databases, articulatory behavior tends to show a lower variation.

## Proposed approach

In this section, the preprocessing and feature extraction routines are first presented, followed by a detailed description of the proposed ACL method.

### Preprocessing

This work focuses on studying peripheral vowels (*/AA/*, */AE/*, */IY/*, and */UW/*) and full-sentence utterances. In this section, we present the approach assuming a vowel-level analysis; however, it is easily extensible to complete utterances as seen in the Experimental Results section. For the peripheral vowel analysis, vowel duration is appropriately chosen as the unit of analysis. The utterances are forced-aligned to obtain the vowel boundaries. For USC EMA, the SailAlign [50] tool was used to perform this task. Forced alignment was not necessary for USC IEMOCAP as boundary information was already provided with the database.

Acoustic, low-level descriptors (LLD) such as energy of 26 MFBs, pitch, first two formants, and overall intensity (30 LLDs) are extracted using sliding and overlapping windows over the vowel segments with a frame rate of 200 frames/s. The overall intensity and energy in MFBs were extracted using the openEAR toolkit [25]. Pitch and formants were calculated using Praat [51]. Five statistics including the mean, standard deviation, minimum, maximum, and range are calculated from each LLD trajectory. This process results in a 150-dimensional feature vector for each vowel segment. The supra-segmental, acoustic features for the *i*^*th*^ vowel segment is denoted by *x*_*i*_.

A supra-segmental representation, similar to the acoustic features, is also extracted from the articulatory targets; we use the mean value of each articulatory target calculated over the vowel segment. The *k*^*th*^ articulatory target of the *i*^*th*^ vowel segment is denoted by $a_{i}^{k}$.

Each vowel segment is assigned the same emotion that external evaluators assigned to the complete utterance during the preparation of the database [13, 47]. In case of binary classification, the four emotions are split into two categories depending on the task under consideration—arousal (happy/angry vs neutral/sad) and valence (happy/neutral vs angry/sad). We chose to binarize the emotion labels as this would allow for evaluation across corpora when annotations for different corpora are not the same. A similar label combination strategy for acoustic emotion recognition was also used in previous studies for the same reason [52, 53]. The emotion label for the *i*^*th*^ vowel segment is denoted by *y*_*i*_.

### L1-regularized logistic regression

The traditional cross-entropy cost function for logistic regression over the acoustic features **x** and binary emotion labels *y* for *N* segments is defined as:

1 $$ {\begin{aligned} f(w) \!= \!-\frac{1}{N} [\sum\limits_{i=1}^{N} y_{i} \log \sigma(\mathbf{w}^{T}\mathbf{x}_{i}) + (1\!-y_{i}) \log(1\! - \sigma(\mathbf{w}^{T}\mathbf{x}_{i}))]. \end{aligned}}  $$

Here, the logistic or sigmoid function, *σ*(·), is given as:

2 $$ \sigma(\mathbf{w}^{T}\mathbf{x}_{i}) = \frac{1}{1+e^{-\mathbf{w}^{T}\mathbf{x}_{i}}}.   $$

Training involves learning the optimal weights **w**^⋆^, by minimizing the given cost function. Recognition on new samples involves label assignment by calculating the posterior probability by:

3 $$ p(y=1|x;w) = \sigma(\mathbf{w}^{T}\mathbf{x}).   $$

To avoid over-fitting and learn a sparse weight vector, it is a common practice to modify the cost function to include an additional L1 regularization term,

4 $$ f_{L1}(\mathbf{w},\lambda_{1}) = f(\mathbf{w}) + \lambda_{1} \|\mathbf{w}\|_{1}.   $$

This regularization term makes this method suitable for feature selection and shows good generalization on high-dimensional feature sets [54, 55]. Such L1-regularized problems have been widely used to learn interpretable representations in different tasks including, but not limited to, computer vision [56] and natural language processing [57]. The objective function in Eq. (4) is convex, and a number of generic or application-dependent techniques are available to solve this problem [58–61].

### Articulation constrained learning

In order to improve emotion recognition performance using acoustic and articulatory information, the proposed articulation constrained learning method is constructed by further modifying Eq. (4) to:

5 $$ f_{\text{ACL}} (\mathbf{w},\lambda_{1},\lambda_{2}) = f_{\mathrm{L1}}(\mathbf{w},\lambda_{1}) + \frac{\lambda_{2}}{M} \sum\limits_{j=1}^{M} (a_{j} - \mathbf{w}^{T}\mathbf{x}_{j})^{2},   $$

where *a*_*j*_ is the articulatory target for the *j*^*th*^ vowel segment, and **x**_*j*_ is the corresponding feature vector as described in Section 3.1. An additional regularization term is included to minimize the mean squared error over articulatory target reconstruction. Here, *λ*_1_ controls the sparsity of the solution, while *λ*_2_ controls the importance given to articulatory target reconstruction relative to classification. Thus, the optimal weight vector is learnt by jointly optimizing over two tasks: (i) articulatory target reconstruction and (ii) emotion recognition. The hypothesis is that the correlation between the two tasks is reflected in the weights and leads to an improvement in classification accuracy, especially when there is limited training data.

The proposed ACL regularizer has several important properties. First, note that the term related to articulatory target reconstruction is not required to operate on the same acoustic data that is used for emotion classification, i.e., it allows for the flexibility to jointly optimize over features corresponding to different speakers and belonging to the same or different databases. Hence, ACL is applicable in scenarios where limited articulatory data is available, but acoustic data is available in abundance. Second, in spite of the additional regularization term, the posterior probability calculation remains the same as Eq. (3). Hence, articulatory data is not required during the recognition step, which is an appealing property, owing to the difficult and time-consuming procedures for articulatory data collection. Lastly, the L1 regularization term enforces the weight vector to be sparse, thus providing a shared and interpretable representation of features that contribute towards both tasks.

**Fig. 5:**
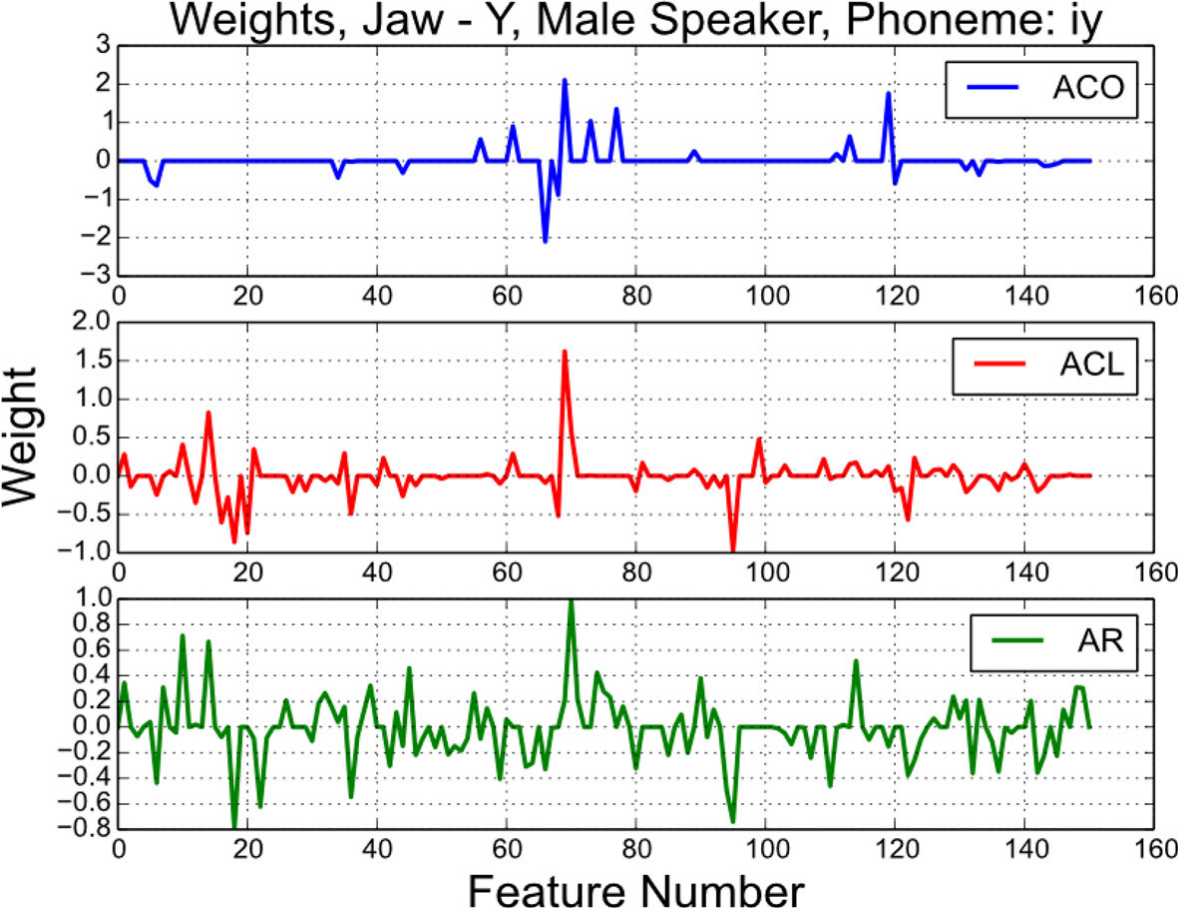
A comparison of weight vectors learnt from different training criteria. (Top row) ACO: emotion recognition using only logistic regression, (middle row) proposed ACL method, and (bottom row) AR: least squares regression over the articulatory target only

A specific example highlighting the last aspect is shown in Fig. 5 and Table 2. Figure 5 shows the weights assigned to each feature for valence classification of the vowel */IY/*. The samples are selected from a single male speaker in the USC EMA database. Three sets of weights are learnt using different objective functions—(i) acoustic only (ACO), which performs emotion recognition using only acoustic features; (ii) ACL; and (iii) articulation only (AR), which performs least squares regression using acoustic features over articulatory targets. In addition to being sparse, the weight vector learnt using ACL captures features relevant to both ACO and AR tasks. For the same example, Table 2 shows the valence classification accuracy as well as the correlation coefficient between the ground truth articulatory target and the reconstruction from acoustic features using the three criteria. In this case, ACL yields a better classification performance over ACO and also performs well over the articulatory target reconstruction task. Ranked on the basis of the magnitude of their weights (as shown in Eq. 5), the top 5 most important features for each training criteria are also shown in Table 2. It can be seen that the top-ranked features for ACL overlap with the top-ranked features for ACO or AR. For instance, ACL shares 3 features with the AR model and 1 feature with the ACO model. This further demonstrates the potential of the proposed ACL method towards feature selection and interpretation, while improving the classification accuracy.

**Table 2:** Example comparison of top-ranked features for different training objective functions

	ACO	ACL	AR
Rank	Top Features		
1	MFB 18, MIN	MFB 18, MIN	MFB 19, MIN
2	MFB 15, MIN	MFB 19, MAX	MFB 19, MEAN
3	MFB 16, RNG	MFB 19, MEAN	MFB 19, MAX
4	MFB 26, MIN	MFB 15, MEAN	MFB 11, MEAN
5	MFB 22, MIN	MFB 21, MEAN	MFB 15, MEAN
UAR (%)	80.1	83.2*	–
CC	− 0.42	0.78	0.90

Results are for valence classification for male speaker and vowel *IY*. Learning is constrained using jaw position along the *Y*-axis, with *λ*_1_=0.5 and *λ*_2_=0.1. *UAR* unweighted average recall, *CC* correlation coefficient, *MFB* Mel filter bank. *Statistically significant improvement (*p* < 0.05) over compared methods

### Extension to multiple targets

The ACL cost function given in Eq. (5) is suitable for learning from a single articulatory target. As described in Sections 2.1 and 2.2, databases often include articulatory information captured from sensors across multiple locations and axes. The proposed modification to ACL for *K* targets is given by a single cost function as:

6 $$ {\begin{aligned} f_{\text{ACL}}(\mathbf{W},\lambda_{1}^{k},\lambda_{2}^{k}) = \sum\limits_{k=1}^{K} [f_{\mathrm{L1}}(\mathbf{w}_{k},\lambda_{1}^{k}) + \frac{\lambda_{2}^{k}}{M} \sum\limits_{j=1}^{M} (a_{j}^{k} - \mathbf{w}_{k}^{T}\mathbf{x}_{j})^{2}], \end{aligned}}  $$

where $a_{j}^{k}$ is the mean value of the *k*th articulatory target for the *j*th vowel segment, and **x**_*j*_ is the corresponding feature vector as described in Section 3.1. Here, the final weight matrix is defined as **W**=[**w**_1_,..,**w**_*k*_,...**w**_*K*_]. Effectively, the function *f*_ACL,*M*_(**W**,·) is a summation of *f*_ACL_(**w**_*k*_,·) over *K* targets. Hence, the learning from each target is considered independently from other targets, i.e., *f*_ACL_(**w**_*k*_,·) is solved independently for each of the *K* targets. During recognition, the posterior probability estimates from the targets are combined to yield an average estimate:

7 $$ p(y=1|\mathbf{x};\mathbf{W}) = \frac{1}{K} \sum\limits_{k=1}^{K} \sigma(\mathbf{w}_{k}^{T}x).   $$

An important benefit of this strategy is that it allows for one to separately measure or investigate the contribution of each target or a group of targets to the overall classification without requiring additional training.

### Extension to multiple classes

So far, the emotion labels were assumed to be binary, arousal, or valence-based attributes. An alternative and intuitive representation for emotions is in terms of discrete or categorical attributes such as happy, sad, and angry. Popular strategies, such as a one-vs-one or a one-vs-rest setup, exist in order to modify the ACL method for recognizing multiple emotion classes. The latter method is adopted here owing to its lower complexity during training. Following this approach, a one-vs-rest classifier is trained for each emotion, i.e., happy vs not happy, and so on. In this case, ACL yields a set of *C* posterior probabilities corresponding to each of the *C* emotion categories. The output label is assigned according to:

8 $$ \hat{y} = \arg\max_{c} p(y=1|\mathbf{x};\mathbf{w}_{c}).   $$

### Optimization

Keeping the regularization coefficients $\lambda _{1}^{k}$ and $\lambda _{2}^{k}$ fixed, the cost functions specified in Eqs. (4), (5), and (6) are convex in *w*. There are several solvers that are suitable for joint logistic and linear regression tasks; a majority of openly available solvers are either applicable to logistic regression or linear regression [60]. In this work, the CVX toolbox for convex optimization was used [61]. Optimization was performed using a splitting conic solver (SCS) with a tolerance level set to 1E−5.

### Selecting the regularization coefficients

Different methods including Bayesian optimization or cross-validation are available to estimate suitable values for $\lambda _{1}^{k}$ and $\lambda _{2}^{k}$. In this work, an exhaustive search across discrete combinations of $\lambda _{1}^{k}$ and $\lambda _{2}^{k}$ is combined with cross-validation. The search is restricted to the following values: $\lambda _{1}^{k} \in \{0.1,0.5,1.0\}$ and $\lambda _{2}^{k} \in \{0.001,0.005,0.01,0.05,0.1\}$. Further details regarding the cross-validation procedures specific to each database are described in Section 4.

## Experimental results: within-corpus

In this section, we describe experiments conducted to evaluate performance in a within-corpus scenario. Here, the acoustic and articulation feature vectors used for ACL belong to the same corpus. Furthermore, the feature vectors used for articulatory reconstruction are speaker-independent from the ones used for emotion classification. For the latter, both speaker-dependent and speaker-independent emotion recognition are considered. A classifier learnt using *ℓ*_1_-regularized logistic regression, i.e., Eq. (4), and an RBF-kernel-based support vector machine (SVM) using only acoustic features, which has been used in previous studies for both binary and multi-class acoustic emotion recognition [52, 53], are considered as the baseline. The unweighted average recall (UAR), which is equal to the average class-wise accuracy, is used to evaluate and compare different methods. Statistical significance is calculated using a difference of proportions test at a significance level of *α*=0.05.

### The USC EMA database (binary classification)

Experiments are carried out using a leave-one-speaker-out (LOSO) strategy. Hence, for the 3 speakers in this database, if the first speaker is designated as the test speaker, then the data from the remaining speakers is used for articulatory reconstruction, i.e., speaker-independent. In comparison, the emotion classification part in ACL is speaker-dependent: a random subset of data belonging to the test speaker is used for training the classifier and the remaining data is used for evaluation. The train/test ratio is kept fixed at 0.5 for all speakers and vowels. This approach is justified as there are only 3 speakers in the USC EMA database, which is not sufficient to obtain reliable speaker-independent emotion classification models.

Given the limited amount of data, the cross-validation procedure for choosing the regularization coefficients $\lambda _{1}^{k}$ and $\lambda _{2}^{k}$ is as follows: First, 15 independent trials of training with different train/test partitions are performed according to the process outlined above. A discrete combinatorial search is then performed to find the coefficients that achieve the best average performance over these trials. The training process is then repeated an additional 10 times over different train/test partitions, but with the selected coefficients only. The recall performance achieved on the test set in each of the 10 trials is then averaged. This entire process is repeated for each vowel and speaker in the database. The final results are presented separately for each vowel, but aggregated across all speakers.

The UA recall performance for binary, arousal, and valence classification tasks are presented in Table 3. Acoustic features respond strongly to changes in emotional intensity; hence, the UA recall for arousal classification is expected to be higher than valence classification. The proposed ACL method does not yield any significant improvements for arousal classification, partly due to this saturation in performance. In comparison, the contribution of ACL is higher towards valence classification. For all vowels except */AE/*, an improvement over the baseline ACO method is observed. Specifically, for vowels */IY/* and */UW/*, the improvement is statistically significant over the baseline ACO method, with a relative increase of ∼ 4%. For full utterances, ACL yields a relative improvement of 2.4% over the baseline ACO method. Similar improvements can also be observed over the baseline SVM method as well. Previous studies have shown that valence classification using only acoustic features is quite challenging, often requiring additional information from alternative sources, such as facial expressions [18, 36]. Articulatory information, which closely relates to facial expressions, likely accounts for the observed improvements for valence classification.

**Table 3:** Within-corpus results for binary classification on the USC EMA database

Vowel	SVM	ACO	ACL	Best target	Best group	BCUAR
Arousal						
*/AA/*	95.26	95.12	93.46	93.84 (JAW, VEL, Y)	93.51 (JAW)	54.51
*/AE/*	91.09	91.47	91.88	91.74 (LIP, POS, X)	92.01 (LIP)	53.16
*/IY/*	95.34	95.59	95.74	95.57 (JAW, POS, X)	95.80 (JAW)	53.35
*/UW/*	94.49	93.64	93.85	93.64 (TNG, ACC, X)	94.41 (LIP)	57.75
*FULL*	99.51	99.51	99.13	99.64 (LIP, POS, X)	99.25 (TNG)	52.88
Valence						
*/AA/*	78.38	77.89	79.46*	79.38 (JAW, ACC, Y)	79.34 (LIP)	53.17
*/AE/*	76.75	78.40	78.34	78.26 (TNG, VEL, X)	78.20 (JAW)	52.47
*/IY/*	70.14	69.45	73.04*	73.90 (LIP, VEL, Y)	73.01 (LIP)	54.02
*/UW/*	76.25	72.01	76.13*	77.63 (JAW, ACC, X)	76.30 (LIP)	53.44
*FULL*	92.85	92.79	95.11*	95.33 (TNG, VEL, X)	95.31 (TNG)	52.68

The UAR is expressed in percentage. The columns of the table represent results from several models: support vector machine (SVM), acoustic only model (ACO), articulation constrained learning (ACL), best target using ACL, best group of targets using ACL, the by-chance unweighted average recall. *Statistically significant improvement(p<0.05) over compared methods

The results above are for the case where the posterior estimates from all the articulatory targets are combined according to Eq. (7) for prediction. It is also possible to obtain estimates from a group of targets or a single target using Eq. (5). The individual targets and groups are defined according to Table 1. For example, the JAW group consists of 6 targets—jaw position, velocity, and acceleration along the *X* and *Y* axes. Here, Table 3 shows both, the UA recall achieved by the best individual target and group of targets and the by-chance UAR (BCUAR). To determine the best targets for USC EMA corpus, instead of learning a single model using all the 18 articulatory targets as described in Table 1, 18 separate models are learnt, one for each articulatory target. Similarly, for the best group, targets are grouped as per the location column in Table 1, yielding 3 groups and their corresponding models. The model with the best performance is selected as the best performing target or group. Between the 3 groups of targets, information from the jaw or lip is found to be more useful on peripheral vowels, while the tongue information is more useful over full utterances. Marginal improvements are observed for arousal and valence classification of */UW/* if only the information pertaining to lip is used for learning.

### The USC EMA database (multi-class classification)

For multi-class or categorical emotion recognition, the training and cross-validation procedures are the same as described earlier for the binary classification case. Again, the articulatory reconstruction is speaker-independent, while emotion classification is speaker-dependent. The recall performance averaged across all 3 speakers for different vowels is shown in Table 4. ACL shows an improvement over the baseline ACO method across all vowels and full utterances. Emotions in */IY/* are the hardest to recognize among all vowels, perhaps due to the saturation in articulatory movement during their expression. These observations are quite similar to the findings in an earlier study on the same database [13]. However, a direct comparison is not feasible as experiments in the latter work were restricted to a single subject and the partitioning strategy for training and testing was not clearly specified.

**Table 4:** Within-corpus results for categorical classification on the USC EMA database

Vowel	SVM	ACO	ACL	Best target	Best group	BCUAR
*/AA/*	80.92	79.97	81.55*	81.17 (TNG, ACC, X)	81.75 (JAW)	28.09
*/AE/*	76.19	78.20	79.02	78.55 (LIP, VEL, X)	79.17 (LIP)	27.35
*/IY/*	71.78	71.15	72.89*	74.66 (JAW, VEL, X)	73.14 (LIP)	27.85
*/UW/*	77.82	75.17	77.50*	76.31 (JAW, ACC, X)	77.80 (LIP)	29.74
*FULL*	95.74	95.62	97.05*	96.97 (LIP, VEL, Y)	97.00 (TNG)	27.23

The UAR is expressed in percentage. The columns of the table represent results from several models: support vector machine (SVM), acoustic-only model (ACO), articulation constrained learning (ACL), best target using ACL, best group of targets using ACL, and the by-chance unweighted average recall (BCUAR). *Statistically significant improvement (*p* < 0.05) over compared methods

Once again, the recall achieved using individual or a group of targets is also shown in Table 4. The experiments for results based on the best individual target and group is the same as those in the binary classification experiments. The jaw and lip sensors are more valuable for categorical classification over peripheral vowels, and the tongue information is useful over full utterances. For instance, the recall performance on vowel */IY/* increases from 72.89 to 74.66% if only the jaw velocity along the *X*-axis is used to constrain learning.

Further inferences regarding the performance can be drawn from the confusion matrices shown in Table 5. Using articulatory information, the accuracy of recognizing happiness across all vowels increases significantly, i.e., from 63.3 to 70.6%. This improvement comes at the expense of a marginal deterioration in recognizing other emotions. For anger and sadness, the accuracy using ACL is 73.5% and 81.3%, respectively, compared to 74.2% and 81.8%, respectively, obtained using the baseline method. Articulatory information also helps to reduce the confusion in discriminating emotions across the valence axis, i.e., between happy-angry and neutral-sad. Here, the rate of misclassifying neutral as sadness is 11.7% for ACL compared to 14.4% for the baseline. Similarly, the rate of misclassifying happy as angry is 16.1% for ACL compared to the baseline 20.4%.

**Table 5:** Confusion matrix for categorical emotion recognition on the USC EMA database

	Neu	Ang	Sad	Hap
(a) ACO				
Neu	79.9	1.9	14.4	3.8
Ang	6.2	74.2	4.1	15.5
Sad	14.4	0.5	81.8	3.3
Hap	8.1	20.4	8.2	63.3
(b) ACL				
Neu	80.8	2.7	11.7	4.8
Ang	6.1	73.5	4.0	16.4
Sad	13.0	1.3	81.3	4.4
Hap	6.0	16.1	7.3	70.6*

Rows represent the ground truth, and columns indicate the recognized emotion. Results are averaged across all speakers and vowels and are expressed in percentage. * Statistically significant improvement (*p* < 0.05) over compared methods

Previous studies on multi-modal emotion recognition also showed that facial expressions, especially those captured from the lower portion of the face, i.e., mouth and chin, are better at recognizing happiness compared to other emotions [36]. Once again, the strong relation between articulatory information and facial expressions likely accounts for the observed increase in performance when we constrain our model to learn acoustic features that model articulation.

### The USC IEMOCAP database (binary classification)

Experiments are carried out using a leave-one-speaker-out (LOSO) strategy for the 10 speakers in this database. Unlike the experiments on the USC EMA database, the acoustic features used for training the emotion classifier and articulatory reconstruction term are completely speaker-independent from the test speaker. Regularization coefficients, $\lambda _{1}^{k}$ and $\lambda _{2}^{k}$, are estimated on the basis of the combination that yields the best average recall over all speakers. Separate models are learnt for each speaker and vowel/utterance, and the final results are presented separately for each vowel/utterance, but aggregated across all speakers.

The UA recall performance for binary, arousal, and valence classification tasks are presented in Table 6. Once again, recall on arousal classification is generally higher than valence classification. Between the two approaches, ACO and ACL, the latter method performs slightly worse or better depending on the vowel. The best improvement is achieved for the vowel */AA/*. For valence classification, ACL outperforms the baseline for each vowel with the largest improvement observed for the vowel */AE/*. For full utterances, marginal improvements are obtained over the baseline ACO method.

**Table 6:** Within-corpus results for binary classification on the USC IEMOCAP database

	SVM	ACO	ACL	Best target	Best group	BCUAR
Arousal						
*/AA/*	65.81	67.43	69.18*	72.60 (LPH, Z)	70.28 (LPH)	65.44
*/AE/*	63.67	64.24	65.40	66.95 (LPH, X)	65.74 (LPH)	63.65
*/IY/*	65.27	66.36	65.85	67.90 (LIP, Y)	67.70 (LIP)	66.22
*/UW/*	62.49	63.90	64.44	67.60 (LPH, Z)	66.50 (LIP)	69.03
*FULL*	72.96	72.58	73.52	75.04 (CHN, X)	75.12 (CHN)	50.47
Valence						
*/AA/*	59.29	60.47	60.68	64.69 (LIP, Z)	63.01 (CHW)	51.21
*/AE/*	57.20	57.61	59.06*	61.49 (LPW, X)	60.02 (CHW)	54.83
*/IY/*	59.61	60.36	61.34	62.85 (CHN, Y)	61.85 (LPW)	56.88
*/UW/*	61.20	63.17	63.62	64.42 (CHW, X)	63.95 (LIP)	56.25
*FULL*	60.34	61.74	62.16	63.37 (LPH, Z)	62.95 (LPW)	60.45

The UAR is expressed in percentage. The columns of the table represent results from several models: support vector machine (SVM), acoustic only model (ACO), articulation constrained learning (ACL), best target using ACL, best group of targets using ACL, and the by-chance unweighted average recall (BCUAR). *Statistically significant improvement (*p* < 0.05) over compared methods

The results above are for the case where the posterior estimates from all the articulatory targets are combined using Eq. (7). It is possible to study the performance obtained using individual or a group of targets. For the USC IEMOCAP database, Table 6 defines the possible groups and individual targets. For example, the group lip height (LPH) consists of 3 targets corresponding to the *X*, *Y*, and *Z* axes. The recall obtained by the best individual target and group for different vowels in the USC IEMOCAP database is shown in Table 6. For the USC IEMOCAP corpus, there are 15 possible targets and 5 possible groups based on the attribute column in Table 1. The best target and best group results reported in Table 6 are derived as per the approach described in Section 4.1. Among the different groups of targets, the markers pertaining to the lip are found to be more useful than the chin markers. Articulation constraints using only a single target yields a better performance in comparison with learning from all available targets. For instance, using only the lip height data along the *Z*-axis, statistically significant improvements are obtained for */AA/*. The relative improvement over the baseline for arousal and valence classification is 7.6% and 6.9%, respectively. Similarly, a recall of 61.49% is obtained for valence classification over */AE/*, a relative improvement of 6.7% over the baseline.

### Comparing the two datasets

Based on the experimental results, the proposed ACL seems to improve emotion recognition performance. The impact on arousal classification is less compared to valence classification. Classification along the valence axis using only acoustic features is quite challenging; hence, the results obtained in this work are of importance. The expectation here is that using ACL, a shared representation can be learnt that would not only lead to reliable emotion recognition, but also be able to reconstruct articulatory targets based on the constraints.

**Fig. 6:**
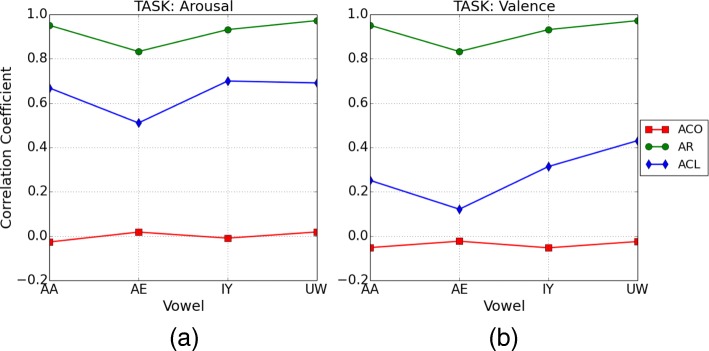
A comparison of the average correlation coefficient over all articulatory targets and speakers under different training criteria for the USC EMA database

**Fig. 7:**
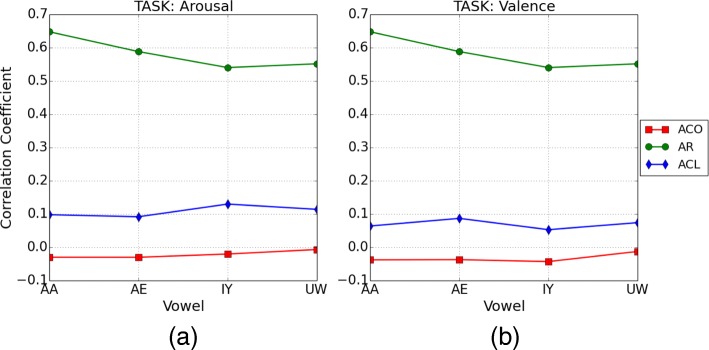
A comparison of the average correlation coefficient over all articulatory targets and speakers under different training criteria for the USC IEMOCAP database

This property is verified through additional experiments, and the corresponding results are shown in Figs. 6 and 7. The correlation coefficient is calculated between the ground truth and reconstructed articulatory targets over all the utterances used during training. This coefficient is calculated for each articulatory target and speaker and the averaged results for each vowel are presented. The reconstructions obtained from the 3 models trained using different criteria (Section 3) are compared: (i) ACO or Eq. (4), (ii) ACL or Eq. (6), and (iii) AR, which is a least squares regression over articulatory targets. As expected, the AR method shows the maximum CC for all vowels as it is optimized for a single task, i.e., articulatory target reconstruction. At the opposite end, the ACO method is bound to perform the worst as articulatory data is not considered at all during training. ACL shows a higher CC than ACO as well as a better emotion recognition performance as seen from our earlier results. Hence, ACL provides an efficient way of incorporating articulatory information to constrain learning, while achieving better generalization and overcoming the necessity of articulatory data collection during testing or recognition.

From the Figs. 6 and 7, a few differences between the USC EMA database and the USC IEMOCAP databases can also be observed. The overall CC on the former is relatively higher compared to the latter. This is due to the fact that the USC IEMOCAP database is larger than the USC EMA database; hence, less regularization is required to improve performance (the *λ*_2_ term is smaller).

In general, the overall recall for emotion classification is lower for the USC IEMOCAP database compared to the USC EMA database. This can mainly be attributed to the difference in expression types across the two databases. Emotions were more naturally expressed in the USC IEMOCAP database; the inter-evaluator agreement for this database measured using the kappa statistic is 0.4 [47]. This metric indicates the difficulty experienced by the evaluators while labeling the utterances, thereby highlighting the naturalness of emotions.

## Experimental results: cross-corpus

It is straightforward to combine data from different domains using the ACL objective function and evaluate performance under more realistic scenarios such as cross-corpus recognition. Here, cross-corpus is with reference to the data used for emotion classification and articulatory reconstruction in ACL. Specifically, the acoustic data used for articulatory target reconstruction belongs to a different database from the one used for training the logistic regression classifier. Experiments are conducted using the same databases, USC EMA and USC IEMOCAP. Recognition performance is evaluated in terms of the UA recall and compared against the within-corpus results obtained in Section 4.

### The USC EMA database

In the first experiment, the USC EMA database is designated as the test corpus. Acoustic features from this database are used to train the logistic regression function in a speaker-dependent manner. Acoustic features and articulatory targets, available from 15 facial markers (Table 1) and all 10 speakers of the USC IEMOCAP database, are used for the constraints enforcing articulatory reconstruction. The training and cross-validation procedures are the same as described in Section 4.1.

The recall performance for arousal and valence classification is presented in Table 7. Overall, there is no significant impact on recall performance in spite of the data being collected from different sources. The deterioration relative to the baseline, within-corpus performance, is severe only for select cases: arousal classification of vowel */IY/* and valence classification of vowel */UW/*. The relative drop in performance is 5% in the former case and 3.4% in the latter case. In a few cases, an improvement in performance is also observed; for instance, valence classification over vowel */AE/*.

**Table 7:** Cross-Corpus, Binary Classification, Test on the USC EMA database

Vowel	ACL (WC)	ACL (CC)	Best group
Arousal			
*/AA/*	93.46	93.85	93.99 (LPP)
*/AE/*	91.88	89.59	90.39 (LPH)
*/IY/*	95.74	90.89	89.90 (LPH)
*/UW/*	93.85	91.04	90.40 (LPH)
*FULL*	99.13	93.84	95.84 (LPP)
Valence			
*/AA/*	79.46	79.65	80.32 (LPW)
*/AE/*	78.34	79.50	79.62 (LPW)
*/IY/*	73.04	73.16	73.42 (CHW)
*/UW/*	76.13	73.51	74.39 (LPH)
*FULL*	92.79	94.92	94.65 (CHW)

### The USC IEMOCAP database

In our second experiment, the USC IEMOCAP database is designated as the test corpus. Hence, acoustic features from this database are used to train the logistic regression model in a speaker-independent manner, while acoustic features and articulatory targets, from all the 18 targets (Table 1) and 3 speakers of the USC EMA database, are used for the term involving articulatory reconstruction constraints. The training and cross-validation procedure is similar to the one outlined in Section 4.3.

The recall performance for arousal and valence classification is presented in Table 8. The impact of cross-corpus training is minimal on recall performance. Similar to the USC EMA database, there is an improvement in recall for select cases: arousal classification for vowel */IY/* and valence classification for vowels */AA/* and */AE/*.

**Table 8:** Cross-corpus, binary classification, and test on the USC IEMOCAP database

Vowel	ACL (WC)	ACL (CC)	Best group
Arousal			
*/AA/*	69.18	68.71	70.11 (TNG)
*/AE/*	65.40	65.01	66.02 (TNG)
*/IY/*	65.85	66.96	67.59 (JAW)
*/UW/*	64.44	63.55	65.57 (TNG)
*FULL*	73.52	74.46	74.97 (JAW)
Valence			
*/AA/*	60.68	62.41	62.80 (TNG)
*/AE/*	59.06	60.83	61.92 (TNG)
*/IY/*	61.34	61.27	61.28 (JAW)
*/UW/*	63.62	63.02	63.23 (JAW)
*FULL*	62.16	61.01	61.42 (JAW)

Based on the above experiments and results, cross-corpus constrained learning is not severely detrimental to the emotion recognition performance. Marginal deteriorations are expected in certain cases as there are notable differences across different corpora [18, 52, 62–64]. These differences exist among the selection of speakers, recording conditions, and types of emotional expressions.

## Discussion

Experimental results in this study show that ACL can improve model performance, even when articulatory information is not available during model evaluation. Within each database, the results are consistent. In within-corpus experiments, we show that our proposed ACL method yields a larger improvement on valence classification. This is consistent with other studies that show classification along the valence axis using only acoustic features is challenging and multi-modal classifiers based on acoustic and articulatory features can improve performance [18, 36]. For multi-class classification, we also see that ACL shows improvement over ACO (without articulatory feature) and the SVM baseline system. Across the two databases (EMA and IEMOCAP), the error patterns are different. This is not surprising since (1) the databases were collected under different conditions, with different speakers and different sample sizes, and (2) different sensors are used to measure the articulator motion, resulting in different articulatory targets. The influence of these factors on acoustic emotion recognition performance was reviewed in [53], where the authors showed that acoustic emotion recognition on different corpora exhibits variable performance due to mismatch of recording conditions, speakers, and types of speech materials. For example, in a binary classification task for arousal (same as ours), the performance of the model varied between 55.0 and 96.8%, depending on which corpus the model was evaluated on. Similarly, the model for valence varied between 50.0 and 87.0%. More consistent performance is expected on larger datasets with a more representative distribution of speaker identities, recording conditions, and consistent articulatory features.

Previous studies have shown that the relationship among acoustic features, articulatory features, and emotion classes can be complex and non-linear [37, 39, 65]. Unlike previous studies that rely on pre-trained acoustic-to-articulatory inversion models for improving the performance on other tasks, our aim in this study is not to first reliably estimate articulatory features. Our goal is to use whatever linear relationship exists between the acoustics and the articulatory parameters to constrain the hypothesis class of the emotion recognition model. In this context, the ACL regularizer ensures that the model does not overfit to the emotion data. A case can certainly be made for using a more complex model for both the loss function and the regularizer; however, we decided against this in this paper for two reasons: (1) typically, larger data sets are required to learn more complex models and (2) the resulting model may no longer be convex. Given the limited sample size in the two databases we used, we opted for a linear model that can be reliably trained and understood.

The proposed ACL can be naturally extended to DNN-based multi-task learning with both emotion and articulatory features as the training targets. Furthermore, since articulatory features are recorded at the segment level while emotion labels are recorded at the sentence level, sentence-level recurrent neural networks can be used to predict both emotion labels and articulatory features on different time scales [35]. We expect that more complex models will see consistent improvement with the proposed ACL on larger and more diverse speech emotion corpora.

## Conclusions

An articulation constrained learning method was proposed to perform emotion recognition using both acoustic and articulatory information. A conventional L1-regularized logistic regression cost function was extended to jointly optimize two tasks—(1) emotion classification via logistic regression and (2) articulatory reconstruction via least squares regression. The proposed method was extended to consider constraints from multiple articulatory targets as well as categorical emotion recognition. The two advantages offered by ACL include its simplicity and its flexibility to combine data from different domains without requiring large-scale articulatory data collection.

Experiments were performed to evaluate speaker-dependent as well as speaker-independent emotion recognition performance on two databases, USC EMA and USC IEMOCAP, providing articulatory information in different manners. On the USC EMA database, significant improvements of 5.1% and 5.7% were obtained for valence classification of vowels */IY/* and */UW/*, respectively, while, on the USC IEMOCAP database, an improvement of 2.5% was obtained for the same task on vowel */AE/*. Discriminating across the valence axis is quite challenging using speech; hence, the results obtained in this work demonstrate the importance of articulatory information towards improving the performance on this task. The performance using individual targets was also presented. In this case, an improvement of 6.9% and 6.7% was obtained for valence discrimination of vowels */AA/* and */AE/*, respectively, on the USC IEMOCAP database. These results show that domain knowledge can be incorporated to improve the performance, i.e., if the relationship between articulatory target behaviors and vowels is known beforehand, then other targets can be ignored or given relatively lower importance during the decision-making process.

For categorical emotion recognition on the USC EMA database, ACL was found to improve the overall performance across all four vowels. Incorporating articulatory constraints was shown to significantly improve the rate of recognizing happy emotions; an 11.55% improvement relative to the baseline was observed. The confusion matrices were presented, and it was shown that ACL tends to decrease the misclassification rate between emotions with similar arousal characteristics, i.e., happy-angry or neutral-sad. This observation is supported by the improvement in valence discrimination described above.

Cross-corpus studies were conducted to evaluate the ability of ACL to generalize across different recording conditions, speakers, and expression types. Articulatory data available from one database was used to constrain emotion classification over acoustic features belonging to another database. The performance in this scenario was observed to be almost similar to the within-corpus scenario, except for select cases. The deterioration observed in these cases is a commonly expected behavior inherent to cross-corpus studies.

In this paper, we focus on emotion recognition as the task of interest. Future work will focus on applying ACL to other well-known problems in the speech processing domain, including speech or speaker recognition, to yield a sparse and physically interpretable representation of the underlying acoustic characteristics. Furthermore, while we use the regularizer to constrain a sparse linear regression problem, a similar approach can be used to constrain the solution space of other learning models including deep neural networks, support vector machines, and kernel-based methods.

## Data Availability

The datasets generated and/or analysed during the current study are available here: for EMA: https://sail.usc.edu/ema_web/; for IEMOCAP:.
